# Thermal behaviour of procaine and benzocaine Part II: compatibility study with some pharmaceutical excipients used in solid dosage forms

**DOI:** 10.1186/1752-153X-7-140

**Published:** 2013-08-20

**Authors:** Adriana Fuliaş, Ionuţ Ledeţi, Gabriela Vlase, Călin Popoiu, Alina Hegheş, Mihai Bilanin, Titus Vlase, Dorina Gheorgheosu, Marius Craina, Simona Ardelean, Dumitru Ferechide, Otilia Mărginean, Liana Moş

**Affiliations:** 1 Faculty of Pharmacy, University of Medicine and Pharmacy “Victor Babeş”, Eftimie Murgu Square 2, Timişoara RO-300041, România; 2 Research Centre for Thermal Analysis in Environmental Problems, West University of Timişoara, Pestalozzi Street 16, Timişoara RO-300115, Romania; 3 Faculty of Medicine, University of Medicine and Pharmacy “Victor Babeş”, Eftimie Murgu Square 2, Timişoara RO-300041, Romania; 4 Faculty of Medicine, Pharmacy and Dental Medicine, “Vasile Goldis” Western University of Arad, Henri Coanda Street 31, Arad RO-310429, Romania; 5 University of Medicine and Pharmacy "Carol Davila", Department of Physiology, B-dul Eroilor Sanitari 8, Bucharest RO-050474, Romania

**Keywords:** Procaine, Benzocaine, Excipients, Compatibility studies, TG/DTG/HF

## Abstract

**Background:**

The compatibility study of active substances with excipients finds an important role in the domain of pharmaceutical research, being known the fact that final formulation is the one administered to the patient. In order to evaluate the compatibility between active substance and excipients, different analytical techniques can be used, based on their accuracy, reproducibility and fastness.

**Results:**

Compatibility study of two well-known active substances, procaine and benzocaine, with four commonly used excipients, was carried out employing thermal analysis (TG/DTG/HF) and Fourier Transform Infrared Spectroscopy (UATR-FT-IR). The selected excipients were microcrystalline cellulose, lactose monohydrate, magnesium stearate and talc. Equal proportion of active substance and excipients (*w*/*w*) was utilized in the interaction study. The absolute value of the difference between the melting point peak of active substances and the one corresponding for the active substances in the analysed mixture, as well the absolute value of the difference between the enthalpy of the pure active ingredient melting peak and that of its melting peak in the different analysed mixtures were chosen as indexes of the drug-excipient interaction degree. All the results obtained through thermal analysis were also sustained by FT-IR spectroscopy.

**Conclusions:**

The corroboration of data obtained by thermal analysis with the ones from FT-IR spectroscopy indicated that no interaction occurs between procaine and benzocaine, with microcrystalline cellulose and talc, as well for the benzocaine-lactose mixture. Interactions were confirmed between procaine and benzocaine respectively and magnesium stearate, and for procaine and lactose.

## Introduction

Before or during the development of solid dosage forms, large scale development trials are normally preceded by the evaluation of possible interactions between a drug and different excipients used in the formulation process. Although, excipients are required to be medically inert, physical and chemical interactions with the active substance can occur. The screening of a novel excipient for possible incompatibilities is therefore an absolute requirement [1,2].

The humidity and temperature can generate interactions between a drug and an excipient. The drug-excipient interactions can affect drug dissolution and/or bioavailability or/and their therapeutic efficacy and safety. The mechanisms by which the excipients affect the stability of drug are chemical reactions, sorption of moisture, and/or catalysis. Hence, the choice of excipients by carrying out systemic study is very important.

Thermal analytical methods are used for the determination of physical properties, kinetic properties, polymorphic forms and transitions, product stability, and excipient compatibility [3-5].

DSC, respectively DTA/HF had been shown to be an important method for the preliminary phase of any preformulation study of solid dosage form to quickly detect the interactions by comparing the thermal curves obtained for the active substance, for the excipients, and for their physical mixtures.

From the appearance, shift or disappearance of endothermic/exothermic peaks and/or variations in the corresponding enthalpy values in thermal curves of drug–excipient mixtures, it can be prove the existence of a possible interaction between the active substance and the excipient used.

A number of techniques have been used for screening of drug–excipient mixtures for interactions or incompatibilities including isothermal stress testing and thermal analysis [4,5]. Thermal analysis has the advantage over conventional isothermal stress testing in that long term storage of physical mixtures and chromatographic analysis are not required and only a few milligrams of sample is needed [4,6-8]. However, the technique has been criticized as being inconclusive because the moisture stress testing is usually not included and the temperature ranges, which are used, are not characteristic of normal storage conditions.

Even if it’s considered a “stepping stone” in formulation, compatibility testing provide only an approximate indication for the selection/deselection of excipients, as the final composition of the pharmaceutical formulation is usually different from the one tested in the compatibility study [9,10].

FT-IR spectroscopy is a simple, fast and an accurate technique for the evaluation of changes which occur during active substance - excipient mixing. The use of FT-IR relies in the analysis of spectra, by the disappearance of an absorption band, modification of its intensity or position (shifting to lower/higher wavenumbers). Also, the appearance of new absorption bands is an indisputable argument that suggests an active substance-excipient interaction. The use of spectroscopic methods, such as FTIR can bring valuable information about the mechanism of interaction, by the assignment of the bands that appear or disappear in the mixture’s spectra, comparative to the ones for active substance and excipient. Compared to classic FTIR spectroscopy, where the spectra acquisition is usually carried out on a pressed-dispersion of sample in KBr, UATR-FT-IR technique provides superior data quality combined with high reproducibility and has some advantages including: fastness, as no preparation of the samples is required, neither for the active substance, the excipient or their mixture; it is non-destructive and requires small quantities of sample which can be recovered from the surface of the spectrometer’s crystal after the analysis; Also, it’s accuracy is sustained by the fact that in case of classic FTIR sample preparation, the use of a mechanical press to form the pellet can induce interactions that normally does not occur in the formulation step, despite the fact that the sample is considered diluted due to the presence of KBr [10].

Combining DSC, respectively DTA/HF technique with a technique such as Fourier Transform Infrared (FT-IR) Spectroscopic analysis, as complementary tool, allows for detail understanding, elucidation, and interpretation of potential interactions at the molecular level.

*Procaine* (4-aminobenzoic acid 2-(diethyl-amino)ethyl ester, Novocain) is a local anaesthetic of the ester type which is hydrolysed *in vivo* to produce *para*-aminobenzoic acid, which inhibits the action of sulfonamides. Procaine is absorbed following parenteral administration, and can be used in combination with vasoconstrictors in order to prolong its action. Pharmaceutical administration forms contain usually 0.25 to 0.5% procaine for infiltration anaesthesia, 0.5 to 2.0% for peripheral nerve block and 10% for spinal block [11]. Procaine is used as well in analgesic, geriatric and antiviral formulations [12].

*Benzocaine* (4-aminobenzoic acid ethyl ester) is considered an anaesthetic of “low solubility”. This class of agents can be applied directly on injured skin and ulcerated surfaces, where they remain localized for long periods of time to produce a prolonged anaesthetic action. Benzocaine is incorporated into a large number of topical preparations [11] for local anaesthesia, such as dental sprays, ear medication or in throat anaesthetic lozenges [13] and as a desensitizer in condoms [14].

The present study was undertaken because the compatibility study of the selected active substances, namely procaine (PR) and benzocaine (BZ) and four excipients of common use for sustained release have not been reported earlier, to our knowledge.

For this purpose, simultaneous TG/DSC measurements and UATR-FT-IR spectroscopy measurements were carried out on each of the components, both in the pure form and the corresponding 1:1 (*w*/*w*) physical mixtures. The absolute value of the difference between the melting endothermic peak temperature of pure drug and that in each analysed mixture and the absolute value of the difference between the enthalpy of the pure active ingredient melting peak and that of its melting peak in the different analysed mixtures were chosen as indexes of the drug-excipient interaction degree. As well, the analysis of FT-IR spectra sustains the interaction which took place.

## Experimental

### Materials and samples

The active ingredients, procaine chlorohydrate (PR) and benzocaine (BZ), were obtained from Sigma Chemical Co (lot No. 34D1214) and have an analytical purity. Excipients tested were: magnesium stearate (MS) (*Union Derivan Spain*), talc powder (T) (*Luzenac Pharma Italy*), microcrystalline cellulose (MC) (*ParChem Trading Israel*) and lactose monohydrate (L) (*Grain Processing Corporation USA*). All the compounds have an analytical purity and were used as received, without further purification.

The mixed samples consisted of equal masses of active substance and each excipient. Physical mixtures were prepared in proportion (*w*/*w*) 1:1 (active substance: excipient) by simple mixing of the two substances in an agate mortar with pestle for approximately 5 minutes. The 1:1 (*w*/*w*) ratio was chosen in order to maximize the probability of observing any interaction.

Thermal analysis was completed using a simultaneous TG/DTA instrument from Perkin-Elmer DIAMOND. The experiments were carried out using aluminium crucibles with approximately 7–8 mg of the sample. For determination of the heat effects, the DTA curves (in μV · mg^−1^) were changed with the Heat Flow curves (in mW mg^−1^), so that the peak area corresponds to an energy in J g^−1^ or kJ mol^−1^. The experiments were completed in an air atmosphere at a flow rate of 100 mL min^-1^. These were performed under non-isothermal conditions by increasing temperature from ambient up to 500°C, at a heating rate β = 7°C min^-1^.

The IR spectra were carried out using a Perkin Elmer SPECTRUM 100 device in the range of 4000-600 cm^-1^ on an UATR device, with 16 acquisitions for each spectrum.

In order to evaluate the accuracy of the measurements, three repetitions have been done with this experimental protocol for the samples and the obtained results were comparable.

## Results and discussion

### Thermal behaviour of the two active substances

**Figure 1:**
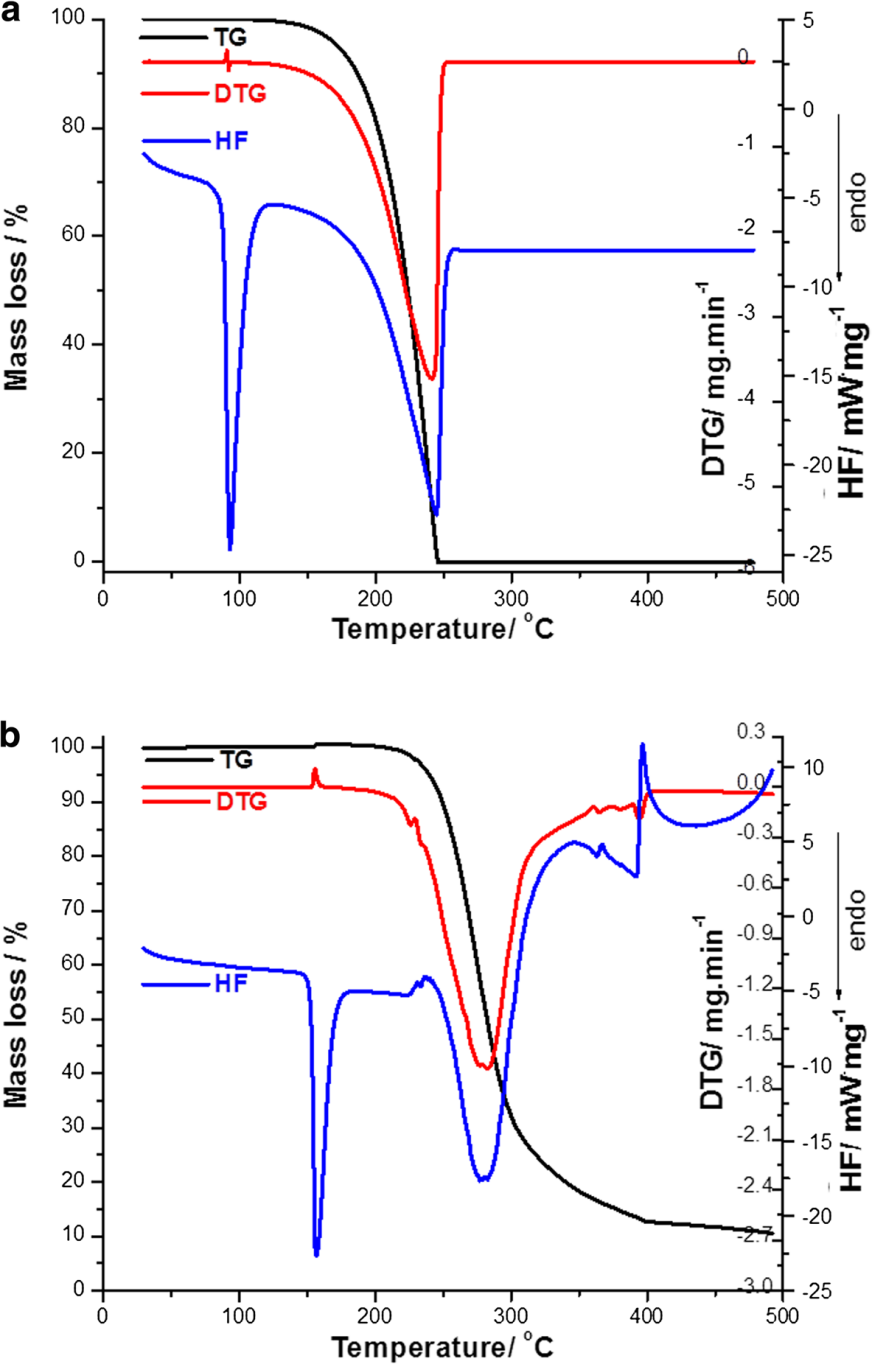
The thermoanalytical curves TG/DTG/HF obtained in air at β = 7°C min^-1^ for the analysed active substances: (a) BZ and (b) PR.

Thermal behaviour of active substances was studied and presented in a previous paper [15]. In order to compare the TG/DTG and HF curves obtained for PR and BZ (active substances) with the ones for active substance-excipient mixture, the first are presented in Figure 1, but at a heating rate β = 7°C min^-1^.

As mentioned in our previous paper [15], thermal decomposition of PR in an air atmosphere occurs in one single process and begins at 196.64°C, as presented in Figure 1. The decomposition occurs in the temperature range 196.6-351.5°C, with the mass loss of 83.57% and *T*_*peak DTG*_ = 278.8°C.

The HF curve of procaine at β = 7°C min^-1^ presents, for beginning, a sharp endothermic peak at 155.8°C (*T*_*onset*_ = 142.8°C; *ΔH*_*fus*_ = 110.462 J·g^-1^) indicating the melting and which corresponds to the values indicated in literature (155-156°C). In this temperature range, it can’t be observed a mass loss on the TG/DTG curves. This process of melting is followed by another two events: the first one is an endothermic process at 279.9°C (*T*_*onset*_ = 238.3°C; *ΔH*_*fus*_ = 243.31 J·g^-1^) and the last one has an exothermic nature with a maximum at 342.6°C (*T*_*onset*_ = 302.8°C; *ΔH*_*fus*_ = −144.79 J·g^-1^).

In the case of benzocaine, the thermal behaviour is similar. The first thermal event for the BZ is an endothermic one, without mass loss, and represents the melting of this active substance (*HF*_*peak*_ = 90.1°C; *T*_*onset*_ = 76.23°C; *ΔH*_*fus*_ = 107.94 J·g^-1^). This value is the same with the melting point mentioned in literature (88-90°C). The following thermal event take place in the temperature range 135.9-247°C with a mass loss percentage Δm = 99.9% (*HF*_*peak*_ = 245.2°C). The experimental determined values for the thermal parameters are similar to the ones obtained in our previous study [15], the differences being attributed to a different heating rate (β = 7°C · min^-1^, instead of β = 10°C · min^-1^) (see Table 1).

**Table 1:** Thermoanalytical data of the two active substances and the used excipients

**Substance**	**TG/DTG curves**		**HF curves**		**Nature of the process**
	**T**_**onset**_**/°C**	**T**_**peak DTG**_**/°C**	**T**_**onset**_**/°C**	**T**_**peak DSC**_**/°C**	
Procaine	194.5	281	148; 240	156; 279	melting; decomposition
Benzocaine	130	241	77; 147	90; 246	melting; decomposition
MC	40; 295	55; 354	50	125	dehydratation; decomposition
L	75; 218	86; 158; 317	254; 367	270; 409	dehydratation; decomposition
MS	50; 280	78; 362	50	117	dehydratation; decomposition
Talc	--	--	--	--	--

### Compatibility study with the excipients by thermal analysis

**Figure 2:**
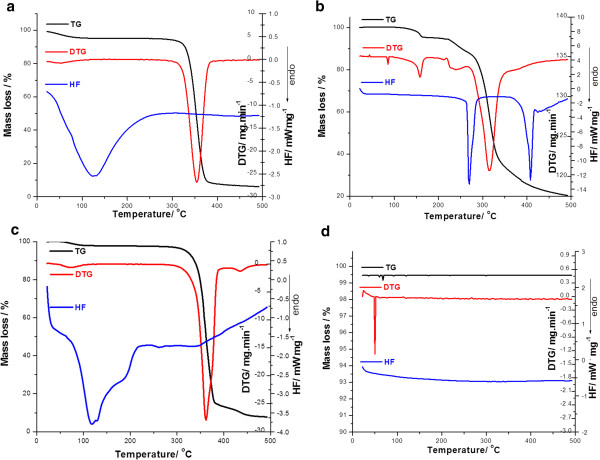
TG/DTG/HF curves in air at 7°C min^–1^ for: (a) magnesium stearate (MS); (b) lactose monohydrate (L); (c) talc (T); (d) microcrystalline cellulose (MC).

The DSC and thermogravimetrical curves of individual excipients which were used in the compatibility study are presented in Figure 2.

**Figure 3:**
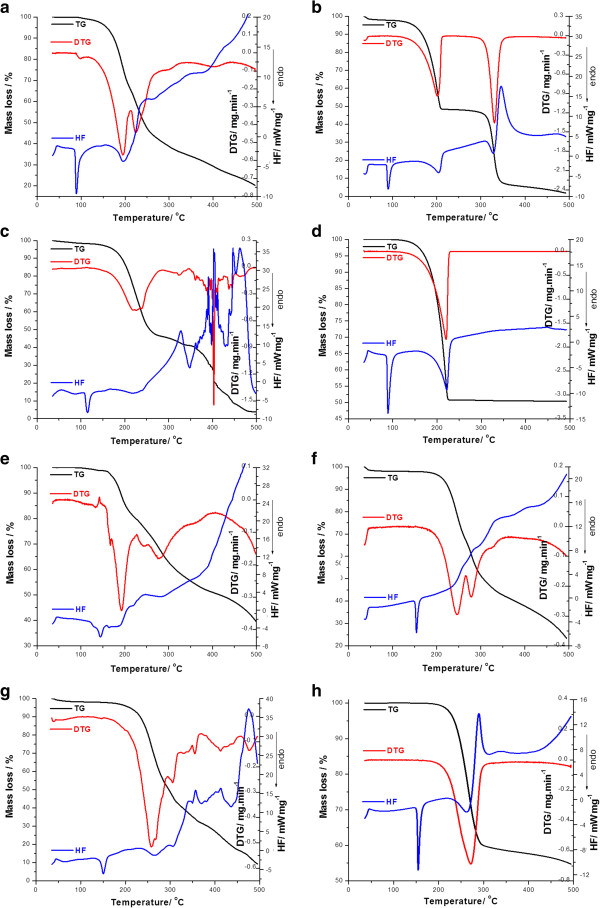
The TG/DTG/HF for the binary mixture of the two active substances (BZ and PR) with different excipients: (a) BZ + L; (b) BZ + MC; (c) BZ + MS; (d) BZ + T; (e) PR + L; (f) PR + MC; (g) PR + MS; (h) PR + T.

The thermoanalytical curves for the analyzed mixtures (binary systems: active substance + excipient) are presented in Figure 3.

The thermal curves of benzocaine and procaine respectively, MC and their physical mixture (Figure 3 (a) and (e)) are almost superimposable. It wasn’t observed any changes in the values of the process’ temperatures (*T*_*onset*_ ot *T*_*peak*_), nor disappearance and appearance of supplementary peaks in the case of the mixtures with MC. The melting behaviour of the drugs (PR and BZ) is not affected by the presence of MC. It may be concluded that there is no interaction between the two active substances and MC under the testing conditions.

Another used excipient is talc, which possess a higher thermal stability, as can be seen in Figure 2 (c). According to V. Balek *et al*. [16], the first mass loss for a sample of talc is observed by TG in the range 900-1050°C and characterized the loss of structural water due to dehydroxylation of Mg_3_Si_4_O_10_(OH)_2_ and accompanied by the formation of enstatite (MgSiO_3_) and silica [17].

As expected, in the thermoanalytical curves of talc, no peaks were observed in the analysed range 50-550°C (Figure 2 (c)). For the two mixtures with this excipient, the endothermic peak which corresponds to the melting process of PR and BZ, respectively was well preserved at 154 and 88°C, respectively in the HF curve of the active substance-talc mixture.

The thermoanalytical curves of magnesium stearate (Figure 2 (a)) present a lot of thermal events. The HF curve of this excipient shows three principal events: the first correspond to the dehydration process from range 75-102°C; the second event (119-130°C) indicates the melting point for palmitic acid salt and stearic acid salt with magnesium, respectively [18], being known that pharmaceutical MS used as excipient is a mixture of different magnesium salts of some fatty acids, the mainly proportions being the salts of stearic and palmitic acid. Being a mixture, this excipient melts in an extent temperature range which can vary between 117 and 150°C, but a high purity form of this excipient was reported having the melting at 128 ± 2°C [19].

The HF curves of benzocaine and procaine, respectively with magnesium stearate reveals a well-defined interaction with this excipient which were evidenced by the shift of the endothermic melting peaks with more than 2°C compared to that of pure active substance.

Due to the comparison of the TG/DTG/HF curves of the two active substances, MS and their binary mixtures, it can be concluded that the stability of the analysed substances (PR and BZ) in these mixtures is changed. Thus, it can be said that this excipient increased the thermal degradation of procaine. The TG curve of the 1:1 (*w*:*w*) binary mixture between PR and MS, shown in Figure 3 (g), when compared to the TG curve of the pure PR and to the TG curve of the pure MS (shown in Figures 1 and 2(a)), confirms the reaction between the drug and the excipient, evidenced by the premature mass loss. The thermal decomposition begins at a lower temperature value (*T*_*onset DTG*_ = 163°C for the mixture with MS) and the maximum of DTG peak takes place at 259°C. Another reason for an accelerated degradation is the value of *Δm* (%) which is greater than 50%, the expected value due to the fact that the mixture is in proportion (*w*/*w*) 1:1. Given these changes, the thermal stability of PR in the presence of MS was reduced, due to the possible chemical interaction between the active substances and excipient under heating. In the case of BZ mixture with MS, the thermal degradation of the active substance begins at a higher temperature value (*T*_*onset DTG*_ = 137°C) and the value for the melting temperature is increased with ≈ 25°C.

The same thing was noticed in the HF curve of the binary mixture of procaine with lactose monohydrate (Figure 3 (f)) that presented a shift of the procaine’ melting peak from 144 to 154°C suggesting a drug-excipient interaction upon mixing and heating treatment. Another difference consists in the fact that the DSC patterns of the binary mixture of PR with lactose monohydrate showed peaks which occurred at different temperature in addition to those recorded for the individual substances (Table 2). It can be seen from this table that in the case of PR-L mixture, an additional prominent DTG peak appeared at *T*_*peak DTG*_ = 191°C (*T*_*onset*_ = 142°C) and the main DTG peak at *T*_*onset*_ = 190°C in the case of pure PR degradation shifted to higher temperature of *T*_*onset*_ = 226°C, probably due to the interaction of lactose monohydrate with this active substance.

**Table 2:** Thermoanalytical data of active substances and their physical mixtures

**Samples**	**HF**		**ΔH**_**fusion**_**/J g**^**–1**^	**DTG**		**Δm /%**
	**T**_**onset (fusion)**_**/°C**	**T**_**peak (fusion)**_**/°C**		**T**_**onset**_**/°C**	**T**_**peak DTG**_**/°C**	
Active substance						
BZ	77;147	90;246	107.95	130	241	99.9
PR	148; 240	156; 279	110.47	194.5	281	90
Drug/excipient						
BZ + MC	84; 161; 309	90; 204; 327	50.23	110	201	55; 45
BZ + L	77; 153	88; 196	48.52	87; 122	99; 195; 226	3; 52
BZ + MS	103	113.5	37.18	137	227	5; 50
BZ + T	78; 159	88; 222	54.70	120	220	50
PR + MC	146	154	50.89	173; 266	247; 280	65
PR + L	114	144	29.35	142; 226	191; 278	50
PR + MS	133	149	32.12	163	259	70
PR + T	144; 219	154; 263; 288	56.78	180	273	45

The values of *T*_*peak*_*, T*_*onset*_ for DTG and HF curves, the mass loss percentage (*Δm*) and the heat of fusion value (*ΔH*_*f*_) of melting event of procaine and benzocaine respectively, in various excipient mixtures, are presented in Table 2.

### Compatibility study with the excipients by UATR-FT-IR spectroscopy

**Figure 4:**
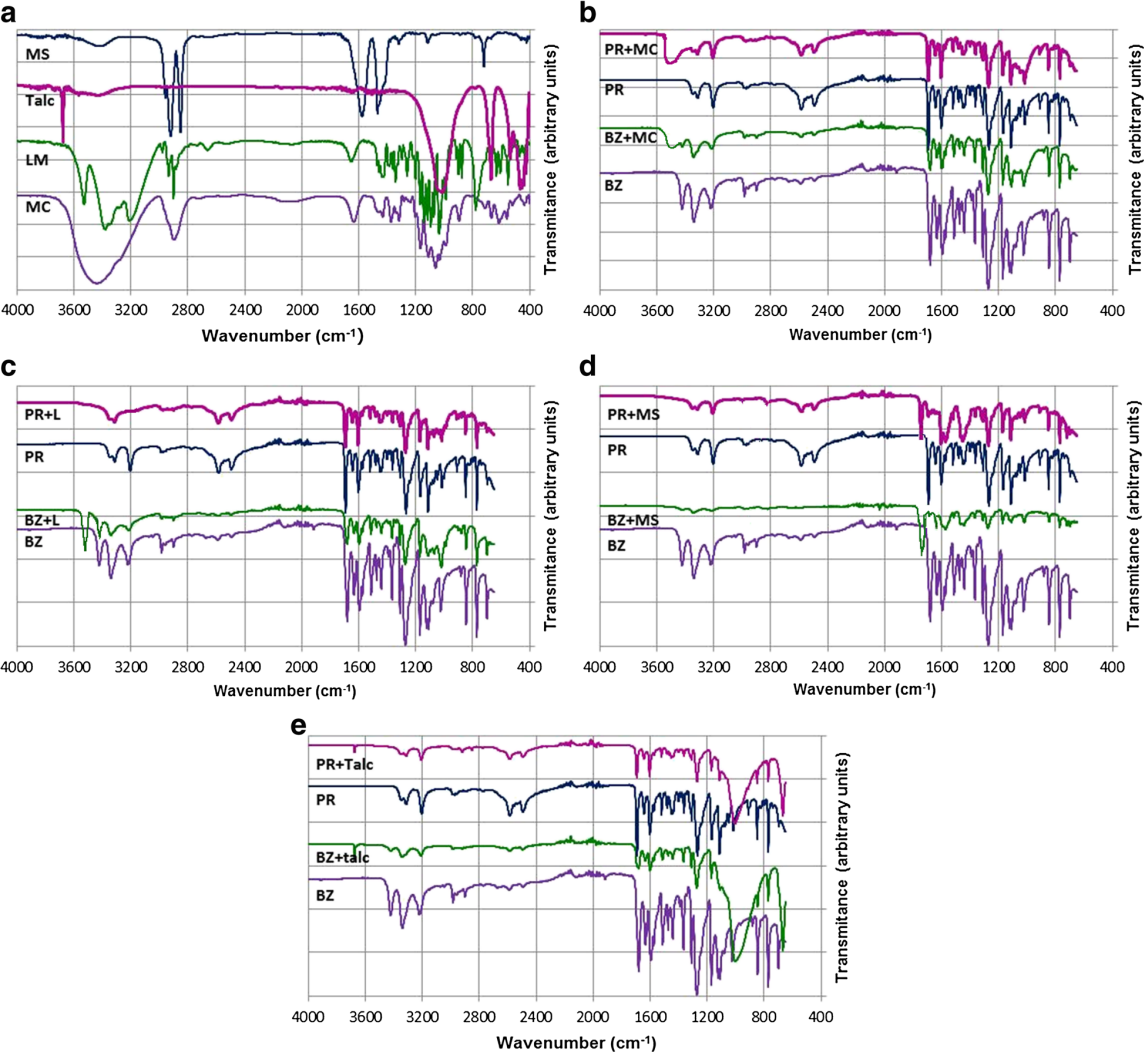
FT-IR spectra of: pure excipients (a); PR and BZ with MC (b); PR and BZ with L (c); PR and BZ with MS (d); PR and BZ with T (e).

The FT-IR spectra of excipients (Figure 4a), and the ones of active substances (PR and BZ) superposed with the studied mixtures (Figure 4b-e) are presented in order to confirm the interaction previously mentioned.

By the analysis of FTIR spectrum of pure PR, one can notice the presence of several distinctive bands, characteristic to vibrational modes of functional groups, as follows: a doublet consisting of two sharp N-H stretching bands at 3345 and 3314 cm^-1^, a –NH_2_ scissoring band at 1604 cm^-1^ and -NH_2_ wagging and twisting bands in the 850-750 cm^-1^ spectral range. The band around 3200 cm^-1^ can be assigned to ammonium ions (due to the fact that procaine was used as a chlorohydrate) The spectrum also shows a C-N stretching band in 1360-1250 cm^-1^ range characteristic for aromatic amines. The presence of a tertiary amino group is sustained by the -N-CH_2_ stretching band around 1170 cm^-1^. The two most polar bonds in esters are the C = O and C-O respectively, which produce distinctive bands in the spectrum around 1700 cm^-1^ and 1200 cm^-1^, respectively. Being an aromatic ester, it is expected that aromatic C = O stretching appears at lower wavenumbers than the ones characteristic for aliphatic ones (which absorb near 1750 cm^-1^), in this case at 1692 cm^-1^. On the other hand, the C-O stretching can be attributed to the intense band around 1250 cm^-1^[20].

The similarity of benzocaines’ structure to the one of procaine lead to the conclusion that IR spectra presents several similarities, namely: a doublet consisting of two sharp N-H stretching bands at 3340 and 3320 cm^-1^, a –NH_2_ scissoring band at 1594 cm^-1^ and NH_2_ waging and twisting bands in the 850-750 cm^-1^ spectral range. The spectrum also shows a C-N stretching band in 1366-1240 cm^-1^ range characteristic for aromatic amines. The C = O and C-O bonds in an aromatic ester produce distinctive bands at 1679 cm^-1^ and 1253 cm^-1^, respectively.

The FTIR bands characteristic to C-C, C-H or aromatic ring does not present interest in our study due to the fact that in formulation step, their breaking is highly improbable. According to this, the analysis of FTIR spectra will be focused on the modification of characteristic bands for N-H and carbonyl groups.

### The analysis of BZ and PR mixtures with MC

Characteristic vibration bands for functional groups (amine, ester) for both active substances can be identified even in the FTIR spectrum of mixtures. No bands suffer modification of intensity or shifting to increased or decreased wavenumbers. The FTIR spectra of PR and BZ mixtures with MC show the presence of the characteristic band for the O-H stretching of hydroxyls from MC at 3450 cm^-1^ as a broad signal. The C = O and C-O bands appear at the same wavenumber as in the case of pure PR and BZ. In this case, FTIR spectroscopy confirms that no interaction occurs (Figure 4b).

### The analysis of BZ and PR mixtures with L

By comparative analysis of FTIR spectrum for BZ and BZ + L, once can notice that no alteration of the spectra occurs. It can be considered that the spectrum of BZ + L mixture is a superposing of both BZ and L spectra, leading to the conclusion that no interaction occurs. On the other hand, surprisingly, the spectrum of PR + L mixture is different than the superposition of the ones for pure compounds. As can be seen, the characteristic O-H stretching from lactose around 3550 cm^-1^ disappear, as well the band characteristic for ammonium ion from procaine (3200 cm^-1^). By the FTIR spectroscopy analysis one can confirm that no interaction occurs between BZ and L, but an incompatibility occurs for PR + L mixture. A tentative explanation for the interaction of procaine with lactose can be attributed to the fact that under heating, procaine hydrochloride decomposes with the release of HCl. It is known that lactose is a disaccharide derived from the condensation of galactose with glucose, by a β-1 → 4 glycosidic linkage. The presence of HCl creates an acidic medium, which can lead to lactose hydrolysis with the formation of the corresponding monosaccharides or their degradation products.

### The analysis of BZ and PR mixtures with MS

MS is the only excipient from the ones’ studied that is incompatible with both active substances. Pure MS presents two intense absorption bands at 1566 cm^-1^ and 1466 cm^-1^, respectively, corresponding to asymmetric stretch of the COO^-^ anion, and at higher wavenumber (2917 cm^-1^ and 2850 cm^-1^) the ones corresponding to vibrations of C-H bonds. The analysis of FTIR spectra lead to the conclusion that the main modification in the molecular structure of both PR and BZ took place at ester carboxyl moiety. In both cases, a significant shifting of bands to higher wavenumbers occurs. In the case of PR + MS, the wavenumber is shifted up to 1722 cm^-1^, and for BZ + MS, is shifted up to 1730 cm^-1^. Literature indicates that stretching of aliphatic C = O occurs in spectral range 1750-1730 cm^-1^[20]. In this case, a possible explanation for this shifting could be the fact that the esteric group suffers a chemical modification, with the transformation of the aromatic ester into an aliphatic one due to the presence of stearate anion or leading to the formation of *p*-aminobenzoic acid, due to the hydrolysis of ester by the presence of HCl. Surprisingly, in the FTIR spectra of mixtures, bands corresponding to COO^-^ anion from MS still appears, leading to the conclusion a part of MS remains unmodified in the mixture.

### The analysis of BZ and PR mixtures with T

For both mixtures, PR + T and BZ + T, the FTIR spectra can be considered as a superposition of the individual ones without modification of bands’ characteristics. The FTIR spectrum of T contains fewer bands than the ones of other excipients, due to the fact that has a simple structure comparative to the ones of organic molecules. The main bands that appear in the FTIR spectrum of T are the ones at 3675 cm^-1^ (stretching vibration of O-H bond from Si-O-H moiety), at 1050 cm^-1^ (stretching vibration of O-Si bond from Si-O-H moiety) and at 675 cm^-1^ and 525 cm^-1^ (asymmetrical vibrations for Si–O bond) [20]. In the case of binary mixtures, the bands appear unmodified compared to pure T. On the other hand, no modifications are noticed for the bands corresponding to PR and BZ. It can be stated that both active substances are compatible with T and no interaction occurs.

## Conclusions

The results obtained from this study confirm that the thermoanalytical methods (TG/DTG/HF) and FTIR spectroscopy can be used as simple and accurate methods in the evaluation of the compatibility between PR and BZ respectively and the four tested excipients.

No interaction was observed between procaine and benzocaine, with microcrystalline cellulose and talc, as well for the BZ + L mixture. This fact is confirmed by FTIR spectroscopy were nor disappearance or appearance of supplementary bands in the case of the mixtures with MC, T and L. It may be concluded that there is no interaction between the two active substances (PR and BZ) and MC and T under the testing conditions, nor for BZ and L.

From the results of the Heat Flow and thermogravimetrical studies, an interaction was confirmed between PR and BZ respectively and MS, and for PR and L. All these experimental observations are sustained by the use of UATR-FT-IR spectroscopy as a complementary tool for compatibility testing.

## Competing interests

The authors declare that they have no competing interests.

## Authors’ contributions

AF, IL, GV, CP and AH formulated the research idea and planned the experiment. The experimental setup was designed by all co-authors. MB, TV had carried out the collection of data. MC and CP had prepared the figures. AF, IL, GV and AH had processed results and tables and had finalized the manuscript. OM, DG, DF and LM have further commented and reviewed. All authors participated in the article’s design and coordination and helped to draft the manuscript. All authors read and approved the final manuscript.
